# A critical review of the chest CT scans performed to detect asymptomatic synchronous metastasis in new and recurrent breast cancers

**DOI:** 10.1186/s12957-019-1584-x

**Published:** 2019-02-23

**Authors:** Justin James, Melanie Teo, Vivekananda Ramachandran, Michael Law, David Stoney, Michael Cheng

**Affiliations:** 1 Breast and Endocrine Surgery Unit, Maroondah Hospital, Eastern Health, Davey Drive, Ringwood East, Melbourne, VIC 3135 Australia; 2 Faculty of Medicine, Nursing and Health Sciences, Monash University, Melbourne, Australia

## Abstract

**Background:**

Chest computed tomography (CTC) has now replaced chest X-ray (CXR) as the first choice of investigation to stage breast cancers in most centers in Australia. Routine staging is not recommended in early breast cancers (EBCs). This recommendation is based largely on the use of conventional tests like CXR as staging investigations (SIs). We looked at our experience with CTC in detecting asymptomatic synchronous distant metastasis (ASM) in new and recurrent breast cancers (RBCs).

**Materials and methods:**

Breast cancer patients from Eastern Health Breast Unit during the period from January 2012 to March 2016 were included in the study. Cases were grouped into early, advanced, and recurrent breast cancers, and outcome of CTC was assessed in each group. Relative risk of potential risk factors (tumor size, axillary nodal status, presence of lymphovascular invasion and estrogen, and HER2 receptor status) with a positive result in CTC was determined.

**Results:**

Fourteen ASMs were detected from 335 CTCs giving an overall yield of 4% (95% CI 1.89–6.47). The overall false-positive rate was 10% due to 35 indeterminate findings that were found not to be metastases after further tests or observation. Even with selective use, CTCs have a low yield of 2% (95% CI − 0.19–4.19) in EBCs. Advanced breast cancers have a 9% incidence of ASMs. None of the clinically isolated locoregionally recurrent diseases were associated with detectable distant metastasis in CTC. The most common cause of indeterminate findings was small pulmonary nodules.

**Conclusion:**

Even with selective use, CTC has a very low yield in EBCs. Advanced breast cancers can benefit from CTC in their initial evaluation due to the higher yield. Locoregional RBCs were not usually associated with detectable metastasis on CTC. The usefulness of CTC in all stages of breast cancer is further reduced by its high rate of false-positive results.

## Introduction

Breast cancer is the most common non-cutaneous malignancy among women in Australia [1]. With an annual incidence of 112 per 100,000, this poses a major health challenge. Widespread acceptance of routine breast screening has changed the pattern of breast cancer incidence. Early breast cancer (EBC), i.e., stage I or II, contributes to 70% of all new invasive breast cancers in the developed world [2]. Such screening-detected cancers will only have a very low risk of distant metastasis at the time of presentation [3, 4]. A small proportion of newly diagnosed breast cancers are identified as stage III at diagnosis. Modern centers commonly use neoadjuvant chemotherapy (NACT) in such locally advanced stage disease. Clinically obvious distant metastatic disease at the time of presentation (stage IV) is rare. “Staging investigations” (SIs)—investigations to detect distant metastasis when no clinical evidence exists—form an important part of the initial work-up of new breast cancers. Accurate staging helps in prognostication. Detection of asymptomatic synchronous metastasis (ASM) can significantly change treatment decisions [5]. However, whether this will result in any improvement in survival is unclear.

Common sites of breast cancer metastasis are the bone, lung, and liver [6, 7]. Traditionally, chest X-ray (CXR) has been used as a screening tool for the detection of ASM in breast cancer patients [8]. CXR is very specific in this regard. However, it has very low sensitivity and cost-effectiveness [9]. With the widespread availability of CT scans, it has become common practice to use CT scans of the chest (CTC) for this purpose [10, 11]. CTC is found to be more sensitive and can reliably evaluate lung parenchyma, mediastinum, axial skeleton, axillary, supraclavicular, and lower cervical lymph node basins, and part of the liver. However, CTC is reported to have false-positive rates of up to 14% [5]. This low specificity poses a significant challenge in its routine use. False-positive results can result in unnecessary further investigations, delay in treatment, and patient anxiety [12].

The role of routine SIs in breast cancer is debatable. It is reported that routine SIs are unnecessary in EBCs with no symptoms of distant disease [13, 14]. It is worthwhile staging stage III patients and patients selected for NACT [10, 15]. These recommendations are based largely on results from conventional SIs where CXR is used for chest imaging [8, 9, 14, 16–18]. It has been suggested that advanced imaging techniques, like CTC, could detect ASM with sufficient accuracy and thus replace conventional staging methods [10]. Thus far, available results have failed to confirm this view convincingly [5, 15, 19, 20].

Despite very clear recommendations from a number of international bodies, practice in the community is found to be very variable [21]. A substantial number of EBC patients are found to undergo SIs [22, 23]. Often, patient wishes may also prompt a clinician to perform an SI [24].

Recurrence of cancer is seen in up to 10% of cases after breast-conserving surgery within 5 years [25]. Thus, newly identified recurrent disease is a frequent cause for new breast cancer treatment episodes in modern breast cancer services. All new recurrences are evaluated with a full metastatic work-up before initiating treatment.

We have used CTC as an initial imaging tool instead of CXR in the staging work-up of new breast cancers and metastatic work-up of recurrent breast cancers for the last 10–15 years. This study is aimed at reporting our experience with the use of CTC.

## Materials and methods

We identified our source population from a hospital database of patients who underwent breast surgery and multi-disciplinary meeting (MDM) discussions. Study was conducted during the period from January 2012 to March 2016. CTCs performed within 3 months of the date of surgery were considered staging scans if these were not performed to investigate a specific symptom. All such cases were selected for further analysis, and required data were extracted manually from electronic medical records and the imaging results database. We recorded the basic clinicopathological data and results of all CTCs. We recorded the outcome of all CTCs and all further tests resulting from findings on CTCs and their outcome. The following definitions were used to record the outcome: positive result—metastasis confirmed on CTC or subsequent investigations initiated by findings on CTC; negative result—benign and normal results on CTC; and false-positive result—indeterminate results where a metastasis was not confirmed by further tests or observation for a period recommended by the MDM (usually 3 to 6 months). We used histological examination to ascertain the stage of disease in patients who had primary surgery. Patients who received NACT were grouped separately since their final pathological stage did not represent their initial stage of disease at the time of the SI. When SI upstaged cases to stage IV and caused modification or omission of curative surgery, we listed these cases as stage IV and retained them in our study. All recurrent cases are identified based on the presenting site of recurrent disease (ipsilateral or contralateral breast, chest wall, regional or distant nodes). They were included in the study if CTC were performed in the absence of any clinical evidence of metastasis in the chest. We calculated the outcome of staging CTCs for each AJCC stage of primary breast cancers and classified patients into the following three clinically distinct groups:

1. EBC, including stage I and II
2. Advanced breast cancers (ABC), including stage III and IV and patients selected for NACT
3. Recurrent breast cancers (RBC), including all patients who presented with a new breast cancer diagnosis after curative treatment of localized breast cancer

SPSS version 23 (IBM Corp. Released 2015. IBM SPSS Statistics for Windows, Version 23.0. Armonk, NY: IBM Corp.) was used for all statistical analyses.

## Results

We examined 726 individual breast cancer-related episodes that occurred during the study period. Table 1 shows basic clinicopathologic characteristics of the study population. The mean age of the study population was 61 years. Ninety percent of the cases had either primary invasive or recurrent ductal carcinoma in situ (DCIS) or invasive disease. DCIS, lobular carcinoma in situ (LCIS), phyllodes tumor, and various benign breast diseases formed the remaining 10% of examined cases. Regarding invasive disease, 85% were estrogen receptor positive while 13% were human epidermal growth factor receptor 2 (HER2) amplified. Close to 40% of all cases had lymph node involvement.

**Table 1:** Population demographics and histological features reported as mean (SD) or prevalence (percentage)

Features	Mean/prevalence
Age (in years at the time of surgery)	61 (13.1)
Histologic type	
DCIS	58 (8)
IDC	553 (77)
ILC	88 (12)
Others	27 (3)
Clinical stage	
Stage 0 (in situ)^#^	51 (7)
Stage I	224 (31)
Stage II	275 (38)
Stage III	61 (8)
Stage IV	7 (1)
NACT	30 (4)
Recurrence	56 (8)
Benign and others	22 (3)
Histological grade of invasive cancer	
Grade 1	100 (14)
Grade 2	261 (36)
Grade 3	261 (36)
Others	104 (15)
Other histological features*	
Estrogen receptor positive	550 (85)
Progesterone receptor positive	478 (74)
HER2 amplified	83 (13)
LVI positive	135 (21)
Node positive	241 (39)

*DCIS* ductal carcinoma in situ, *LCIS* lobular carcinoma in situ, *IDC* invasive ductal cancer, *ILC* invasive lobular cancer, *NACT* neoadjuvant chemotherapy, *HER2* human epidermal growth factor receptor 2, *LVI* lymphovascular invasion

*These histological features are calculated on available data on invasive disease only

^#^In situ disease is composed of both DCIS and LCIS; nine of the DCIS were recurrent and hence counted under recurrences in clinical stage

**Fig. 1:**
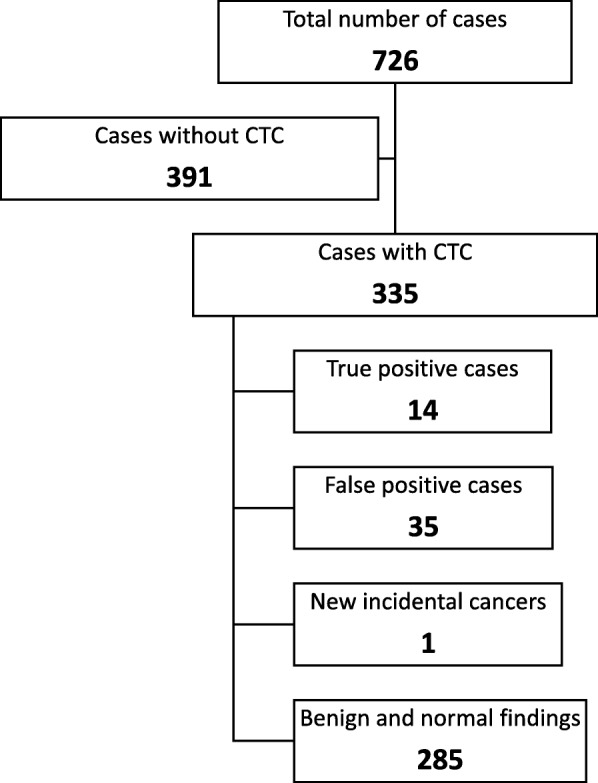
Flowchart of the study population. From 726 breast cancer cases treated during the study period, 335 had available staging CTC data. Fourteen (4%) new metastases and one incidental lung cancer were identified through these scans. Eighty-five percent of all scans only showed benign or normal findings, while 10% had indeterminate findings that were identified as false positive after further tests or observation

Figure 1 is a flowchart showing the course of the study cohort. There were 335 CTCs for study from these patients. Eighty-five percent of all CTCs had no significant finding of concern. Fourteen ASMs and one case of new incidental lung cancer were diagnosed through these scans. Thirty-five of the remaining positive findings were confirmed to be false positives on further testing or observation (Table 2). The overall yield of positive results from these tests was 4% (95% CI 1.89–6.47). The overall rate of false-positive results (FPR) was 10% (95% CI 7.02–13.87).

**Table 2:** Result of CTCs

Final result	*n* (%)
Normal	216 (65)
Clearly benign lesions	69 (21)
Indeterminate lesions requiring further tests	44 (13)
True positive	9 (3)
False positive	35 (10)
Clearly metastatic lesions	5 (2)
Incidental cancers	1 (0.3)
Total number of metastases identified	14 (4)
Total	335

Results of CTC for each clinical group are summarized in Table 3. There were 12 ASMs from 293 CTCs on primary breasts cancers (four in EBCs and eight in ABCs).

**Table 3:** Results of CTC in each clinical stage

Clinical stage	Total number in the study (%)	Number scanned (%)*	True positive (yield %)	False positive (FPR %)	Number needed to scan
EBC	499 (69)	200 (40)	4 (2)	22 (11)	50
ABC	98 (14)	93 (95)	8 (9)	11 (12)	12
RBC	56 (8)	36 (64)	2 (6)	1 (3)	18
Others^#^	73 (10)	6 (2)	0	1	–

*EBC* early breast cancer, *ABC* advanced breast cancer, *RBC* recurrent breast cancer, *FPR* false-positive rate

*Percentage of cases with CTC from the total number of cases at each clinical stage in the study cohort

^#^Others composed of in situ disease, phyllodes tumor breast sarcoma, and cases where no invasive disease was seen on final histology

Tables 4 and 5 summarize the association of known prognostic and predictive factors with the incidence of ASM in primary breast cancers in our cohort. The size of the primary tumor was known for 257 cases (only in cases where primary surgery was performed and data were available). Mean tumor size in cases with metastasis was 38.3 mm, while in non-metastatic cases, it was 29.7 mm. Although the mean tumor size was larger in cases with ASM, this difference was not statistically significant (Table 4). None of the other four known risk factors examined (estrogen and HER2 receptor status, presence of lymphovascular invasion on histology, and presence of metastasis in ipsilateral axillary lymph node) had a statistically significant risk for ASM (Table 5).

**Table 4:** Size of primary tumor and relationship with distant metastasis in patients who had primary resection

Metastasis on SI	*n*	Mean tumor size (mm) (SD, range)
Yes	6*	38.3 (10.3, range 25–50)
No	251	29.7 (18.3, range 3–130)

Difference in mean tumor size is 8.7 mm (95% CI − 6.1–23.5) which was not significant (*p* > 0.05)

*All RBCs and ABCs without primary resection were not included in this analysis

**Table 5:** Potential histological risk factors and their relative risk for positive result on CTC

Histological feature	Number of positive results	Relative risk (*p* value)
ER-negative disease	3	1.3 (0.72)
Presence of LVI	4	1.1 (0.84)
Node-positive status	6	0.91 (0.89)
HER2 amplified	2	0.69 (0.62)

*CTC* CT scan of chest, *ER* estrogen receptor, *LVI* lymphovascular invasion, *HER2*, human epidermal growth factor receptor 2

Four cases of ASM were identified from the 200 cases that presented clinically with EBC, thus giving a yield of 2% (95% CI − 0.19–4.19) in EBCs (Table 4). However, there were 22 cases of false-positive results needing further investigations (FPR = 11%). These results were obtained through the selective use of staging CTC in EBCs, as only 40% of all EBCs treated during this study had SI. Exact indications for staging in EBCS were not easily available from case note review and hence not analyzed in this study. Positive axillary nodal disease and aggressive histological features necessitating adjuvant chemotherapy are some of the common reasons cited for staging work up in EBCs.

The composition of ABC and rates and results of CTC in this group are summarized in Table 6. The probability of a positive finding on CTC was four times higher in ABCs relative to EBCs (Table 4). Other than the advanced stage at presentation, none of the examined risk factors were found to increase the chance of ASM in new breast cancers.

**Table 6:** Composition of ABC and results of each group

Type of ABC	Number scanned (%)	True-positive results (yield %)
Stage III	58 (95)	2 (3)
Stage IV	7 (100)	6 (86)
NACT	28 (93)	0
Total	93 (95)	8 (8)

*ABC* advanced breast cancer, *NACT* neoadjuvant chemotherapy

Recurrent breast cancers (DCIS or invasive disease) formed 8% of all cases in the study (Table 3). The most common form of recurrent disease treated was locoregional recurrence (LRR) (breast, chest wall, or ipsilateral axilla) (Table 7). The results of CTC were available in 36 cases of recurrent disease (nine were recurrent DCIS) (Table 3). A yield of 6% and FPR of 3% were found in the metastatic work-up of our RBCs. Notably, none of the presentations of LRR had a positive CTC.

**Table 7:** Composition of recurrent breast cancers in the study population and its outcome

Primary site of recurrence	Number in the study cohort	Number of available CTCs	True positive	False positive
Breast/chest wall	46	28	0	1
Regional	5	3	0	0
Distant (distant lymph nodes, bone, or liver)	5	5	2	0

**Fig. 2:**
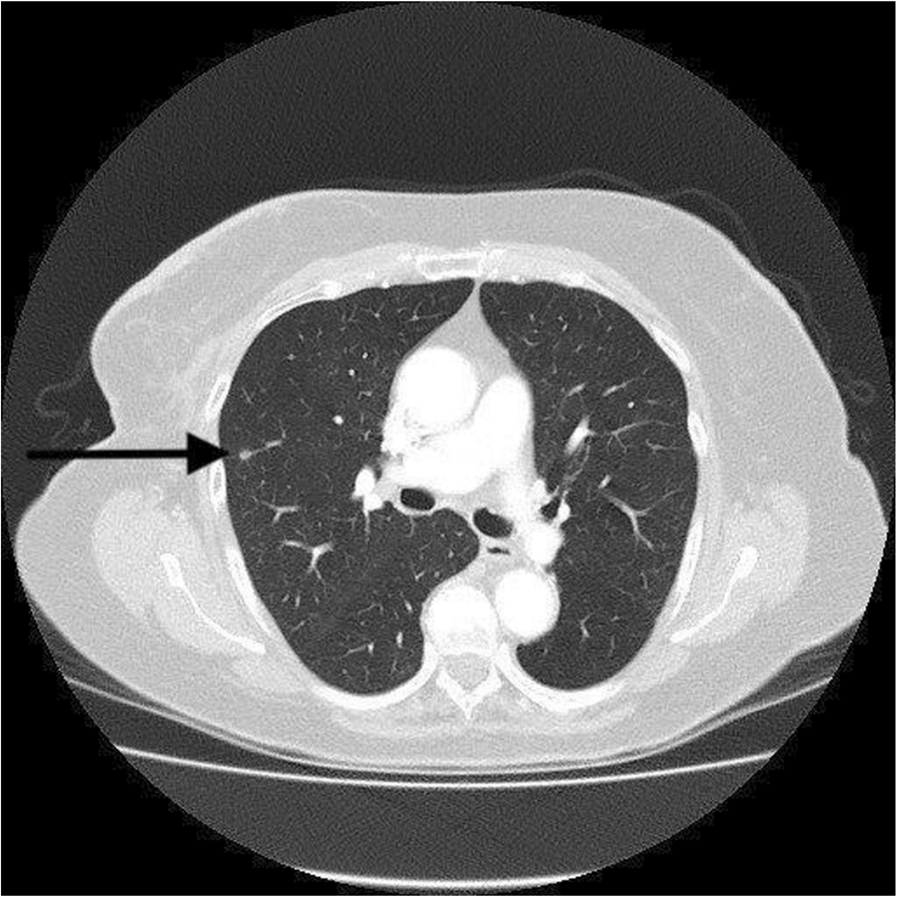
Typical indeterminate pulmonary lesion. The high sensitivity of CTC leads to the identification of very small lesions that would not be obvious on normal chest X-ray. These indeterminate results require further tests or observation to characterize the true nature of these lesions. This results in a high false-positive rate for staging CTC (10%)

**Fig. 3:**
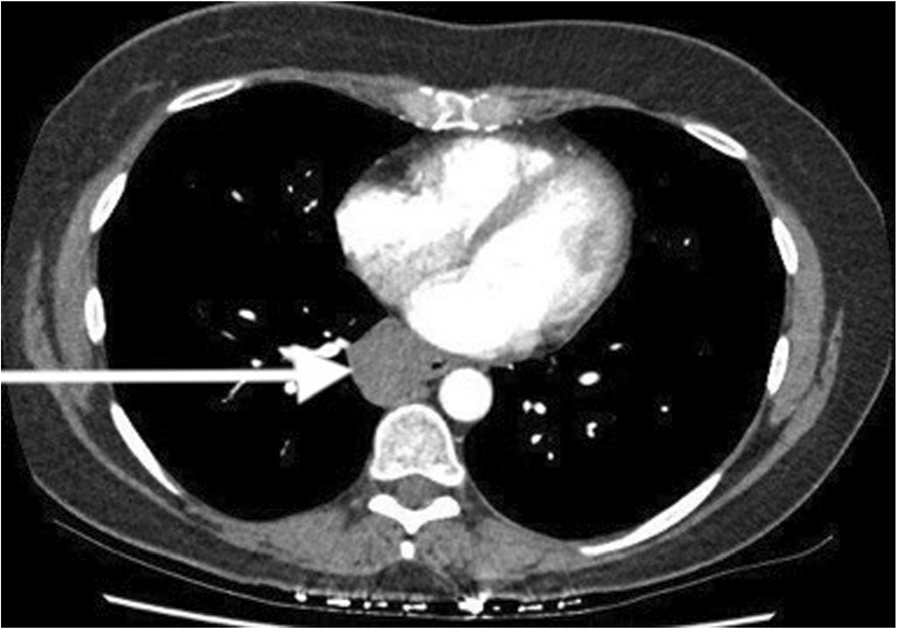
Incidentally found duplication cyst of the esophagus. Incidental lesions can be a cause of false-positive results as in this case. This posterior mediastinal lesion needed required positron emission tomography to characterize its true nature. These types of findings on staging CTC lead to further expensive tests and can be a cause of significant anxiety for patients

Metastases were identified as lung nodules or pleural effusion in 10/14 cases (Table 8). CTC identified metastasis in the bone in nine cases, in non-regional lymph nodes in five cases, and in the liver in one case. One incidental case of lung cancer was also diagnosed. The majority of false-positive results were due to indeterminate pulmonary nodules (74%) (Fig. 2). These are lesions which are too small to characterize radiologically but hard to ignore in the presence of breast cancer diagnosis. Only two of these lesions were biopsied for confirmation of which one was negative, while the second one confirmed primary lung malignancy. Others were watched with serial CTCs. Other lesions that caused false-positive results are listed in Table 9. A mediastinal mass from esophageal duplication cyst was one such false-positive finding (Fig. 3). Two of the mediastinal nodal lesions were biopsied under endobronchial ultrasound guidance. One of these lesions turned out to be true positive, while the second only showed benign changes.

**Table 8:** Type of metastasis identified on CTC

Age at presentation	Clinical stage	Metastasis identified on CTC
48	EBC	Lung
55	EBC	Bone, lymph node
83	EBC	Lung
66	EBC	Lung
34	ABC	Liver, bone
46	ABC	Pleural effusion, bone
41	ABC	Pleural effusion, bone, pericardial effusion
79	ABC	Lung, bone
78	ABC	Pleural effusion, bone
61	ABC	Lung, bone, lymph node
59	ABC	Lung
84	ABC	Pleural effusion
54	RBC	Liver, bone, lymph node
47	RBC	Bone, mediastinal node

*EBC* early breast cancer, *ABC* advanced breast cancer, *RBC* recurrent breast cancer

**Table 9:** Causes of false-positive results on CTC

False-positive finding	*n* (%)
Pulmonary lesions	26 (74)
Mediastinal lesions	7 (20)
Thyroid lesions	4
Nodal lesions	2
Esophageal duplication cyst	1
Neck and axillary lesions	2 (6)
Bone lesions	2 (6)
Total	37

Total exceeds 35 because of 2 cases with 2 different indeterminate findings

## Discussion

EBCs form 70% of new breast cancers treated in modern breast cancer practice. However, the incidence of ASM is very low in EBCs. Current guidelines discourage routine use of SIs in this group of patients. Of the 499 EBCs treated during this period, we performed SIs on only 200 (40%) cases. Linkugel et al. in their recent study noted a scanning rate of 27% among their cases of EBC [20]. Such variation in practice is commonplace as discussed above. In our institution, SIs were performed in EBCs only if they were at high risk of distant metastasis due to aggressive histological features or the presence of lymph node metastasis. We found four cases of ASMs from 200 scanned EBCs (yield = 2%). Kim et al. found only one positive result from 1286 CTCs in EBCs [5]. However, this seems to be from the non-selective use of CTC in EBCs. Bychkovsky’s finding of a 2.1% incidence of ASM in stage II cases from a combination of modern tests (CT scans, liver function tests, and tumor marker assay) is comparable to ours in that only 58% of stage II cases were scanned in their cohort [26]. A study by Linkugel reported a 1.2% yield from a combination of advanced imaging studies (CT scans, bone scans, and positron emission tomography), again in a selected group of EBCs [20]. Our study examined the performance of selective use of CTC as an SI in EBCs as it is used in most centers, and our results prove that even with such selective use, staging CTC has a low yield.

Stage III and IV cancers form only a minority of newly diagnosed breast cancers. Only 14% of all cases studied were advanced stage disease in our study. CTC results were available in 95% of them. ASM is more common at advanced clinical stages [7]. The 9% yield in these cases supports the view that ABCs may undergo CTC for staging. Our result is comparable to those from Piatek et al. (5%) and Kim et al. (6%) [5, 27].

We treated 56 recurrent breast cancers during this period. Some of them may in fact be a second primary rather than true recurrence, since this group included all ipsilateral and contralateral cases as breast recurrence. Within this limitation, from the 31 cases of LRRs, we did not identify a single distant metastasis. It is well known that LRR increases the risk of distant relapse [28]. Our results support the view that LRR and distant recurrences are two independent events [29]. When a patient presents with obvious distant recurrence, they are not candidates for surgical treatment and hence are not well represented in our study. From five cases with clinically obvious distant non-thoracic metastatic disease, CTC identified metastatic spread in the thorax in two.

The advantage of CTC as an SI lies in the fact that it can image the lung, bone, and soft tissues. Ten of fourteen positive cases had lung involvement either in the form of pulmonary nodules or pleural effusion. Bone or intrathoracic nodal involvement was the sole metastatic disease in the remaining four cases.

The high sensitivity of CTC led to a false-positive result in 10% of scans. Kim et al. reported false-positive findings in 14% of 1703 CTCs [5]. Bychkovsky et al. reported that one third of all torso scans needed further tests or observation because of indeterminate findings [26]. The most common cause for a false-positive result is an indeterminate pulmonary nodule (Fig. 2). One case of incidental new lung cancer was also detected. One of the mediastinal lesions turned out to be an esophageal duplication cyst (Fig. 3). These false-positive results required further investigations including CTC, MRI, positron emission tomography, ultrasound, and invasive biopsies to characterize these lesions. These investigations incur further expense and cause significant anxiety to the patient concerned. This factor needs to be considered when ordering these investigations in low yield situations, like in EBCs.

Our study is limited by its retrospective nature. We could only calculate yield and false-positive rate for CTC. The utility of the test also depends on the false-negative rate, which requires follow-up data, as our data is currently insufficient to calculate false-negative rates. Furthermore, CTC is combined with CT abdomen and pelvis and bone scan in most of these cases. Therefore, decisions are often influenced by the findings in these tests. We did not attempt cost calculations because of this.

## Conclusions

CTC has become one of the investigations of choice for staging and metastatic work-up of new and recurrent breast cancers. Staging is performed selectively in EBCs. Even with such selective use, the yield is low with frequent false-positive results and hence cannot be recommended. A more selective use seems to be appropriate in EBCs. CTCs have a better yield among cases of ABC suggesting this may be routinely performed in ABCs. Clinically isolated LRR is unlikely to be associated with a positive result on CTC.

## Abbreviations

ABC: Advanced breast cancer
ASM: Asymptomatic synchronous metastasis
CT: Computed tomography
CTC: Computed tomography chest
CXR: Chest X-ray
DCIS: Ductal carcinoma in situ
EBC: Early breast cancer
ER: Estrogen receptor
FPR: False-positive rate
HER2: Human epidermal growth factor receptor 2
IDC: Invasive ductal cancer
ILC: Invasive lobular cancer
LCIS: Lobular carcinoma in situ
LVI: Lymphovascular invasion
MDM: Multi-disciplinary meeting
NACT: Neoadjuvant chemotherapy
RBC: Recurrent breast cancer
SI: Staging investigation
